# Celebrating 20 years of human genomics: a journey of discovery

**DOI:** 10.1186/s40246-023-00569-2

**Published:** 2024-01-02

**Authors:** Vasilis Vasiliou

**Affiliations:** Department of Environmental Health Sciences, Yale School of Public Health, New Haven, USA

Two decades ago, Henry Stuart Publications launched the journal *Human Genomics*. Its enduring goal remains to promote and review genomics approaches that address key questions in biomedical research [1]. In 2012, the journal moved to BMC as its publisher, turning it into an open-access, fully online publication ensuring the published research would be shared as broadly as possible [2]. To this day, *Human Genomics* continues to focus on applying genomic and epigenomic analyses to understand complex human diseases and traits, especially at the genomic level. In 2015, it became the official journal of the Human Genome Organization (HUGO). This year, we proudly celebrate *Human Genomics*' remarkable two-decade journey. Since its inception, the journal has been significantly contributing to our understanding of the human genome's complexities and its impact on medicine, genetics, human health, and beyond. *Human Genomics* remains focused on advancing basic research into the human genome and human normal and disease phenotypes.

Today, supported by an international Editorial Board of experts, the journal covers a wide range of topics in human genomics, including pharmacogenomics, genome-wide studies, sequencing, functional genomics, epigenomics, and much more, especially basic research. *Human Genomics'* strength lies in its ability to address technological advances, from the Human Genome Project to precision medicine, CRISPR-Cas9 gene editing, and the COVID-19 pandemic. It has consistently provided a platform for pioneering work, not only documenting genomics progress but also shaping the field's direction.

Over the past two decades, the journal has played a vital role in fostering a dynamic platform for researchers, scientists, and clinicians to share groundbreaking findings, innovative methodologies, and insightful perspectives. It has been a cornerstone of the human genomics community, promoting collaborative research and facilitating knowledge dissemination that has transformed our understanding of human genetics on topics covering population genetics, genetic variation, disease genetics, pharmacogenomics, and ethical considerations. This comprehensive approach bridges the gap between basic research and real-world applications, integrating genomics into clinical practice.

Following are some particularly noteworthy accomplishments of the journal over the last two decades:

*Gene Families*: In the fast-paced world of genomics research, staying updated on the latest gene family developments is crucial for scientists and clinicians, especially if they are not experts in this area. *Human Genomics* introduced the successful Gene Family Updates series which offers comprehensive, up-to-date reviews of select gene families. The identification by Dan Nebert of the value of (and advocacy for) gene names based on amino acid sequence homologies and evolutionary divergence has formed a foundation for the nomenclature of gene families.

*Software Reviews*: *Human Genomics* features a highly impactful series of software reviews. Plans for the future include extending this series to explore how advanced analytics, deep machine learning, and artificial intelligence can promote genomics advancements.

*Genomics of COVID-19*: SARS-CoV-2 and COVID-19 papers published in the *Human Genomics* have advanced our understanding of the virus, its transmission, and its interactions with the human genome that influence human susceptibility to disease. These contributions have informed public health measures, vaccine development, and treatment strategies. As the pandemic evolves into the endemic phase and new challenges emerge, *Human Genomics* remains dedicated to providing a platform for rigorous and innovative research that shapes our response to SARS-CoV-2 and future pandemic health crises.

*Special Issues*: *Human Genomics* is renowned for its commitment to advancing the field of genomics through its publication of special issues that delve into cutting-edge topics and emerging trends. These issues serve as curated platforms, bringing together a collection of in-depth articles, reviews, and research findings that explore specific themes within human genomics. With a focus on fostering interdisciplinary collaboration, the special issues foster the dissemination of knowledge, and promote a deeper understanding of the complexities and implications of genomic research for human health and disease. Through these publications, the journal plays a crucial role in shaping the discourse and pushing the boundaries of genomic science.

*Human Genomics* has consistently maintained rigorous editorial standards, ensuring that published research meets the highest quality and relevance criteria. The dedication of its editorial board and expert reviewers has been pivotal in upholding the journal's reputation for excellence.

This is reflected in the impact factor of *Human Genomics* rising throughout the years to almost 5 in 2023.

While we are proud of the accomplishments of *Human Genomics* over the past 20 years, we anticipate the coming years will hold the promise of even greater genomics advancements, particularly as they relate to personalized medicine, gene therapies, and a deeper understanding of the genetic basis of complex diseases. The journal will remain a beacon of excellence, guiding curious minds through these transformative times and helping usher in an era of unprecedented innovation and discovery.

In conclusion, *Human Genomics* has been a steadfast companion on the journey of unraveling the secrets of the human genome. Its enduring commitment to excellence, adaptability, and scholarly rigor has made it an indispensable resource in the human genomics community. As we celebrate its twentieth anniversary, let us look forward to the continued contributions and insights the journal will undoubtedly bring to the world of human genomics in the years to come.
